# The cavernous carotid inferolateral trunk and persistent primitive maxillary arteries: analysis of dissected arterially injected fetal specimens and high-resolution micro-CT of the dog’s anastomotic arteries

**DOI:** 10.1007/s00276-021-02778-7

**Published:** 2021-06-06

**Authors:** E. Leon Kier, Gerald J. Conlogue, Lawrence H. Staib

**Affiliations:** 1 Yale University School of Medicine, PO Box 208042, New Haven, CT 06520-8042 USA; 2 Quinnipiac University, 275 Mount Carmel Ave, Hamden, CT 06518 USA; 3 Yale University School of Medicine, P.O. Box 208042, New Haven, CT 06520-8042 USA

**Keywords:** Anatomy, Comparative, Humans, Fetus, Dissection, Dogs, Carotid arteries

## Abstract

**Purpose:**

The presence of a persistent primitive maxillary artery is described in the literature dealing with the development of the cavernous carotid inferolateral trunk, and the relevant similarities of the cranial circulation of the human and dog. The literature includes no dissection photographs of the above-mentioned two human fetal arteries, only diagrammatic representations. This study’s objectives were to analyze photographs of fetal dissections for the presence of these two arteries, and also investigate the possibility of obtaining, in preserved dog specimens, high-resolution micro-CT imaging of arteries homologous with the above-mentioned two human arteries.

**Methods:**

The literature describing the embryologic development of the cavernous carotid inferolateral trunk, the persistent primitive maxillary arteries, and their homologies in the dog was reviewed. Relevant dissections of fetal specimens were analyzed. High-resolution micro-CT images of un-dissected dog arteries were produced and analyzed.

**Results:**

Photographs of fetal specimen dissections demonstrate the cavernous carotid inferolateral trunk. A separate persistent primitive maxillary artery was not present in the dissected specimens. High-resolution micro-CT images of the dog demonstrate homologous arteries with segments of the human inferolateral trunk, and other skull base and brain arteries.

**Conclusion:**

This investigation provides the only photographs in the literature of dissected human fetal cavernous carotid inferolateral trunks. A persistent primitive maxillary artery was not present in the dissected specimens and is a non-existent structure, likely a previously misidentified carotid inferolateral trunk. High-resolution micro-CT images of the dog visualized arteries that are homologous to segments of the human cavernous carotid inferolateral trunk artery.

## Introduction

The complex arterial anastomoses of the human orbitocranial region have been studied extensively, stressing the number of dangerous interventional anastomoses. Super-selective interventional neuro-angiography highlighted the potential dangerous complications of intra-arterial therapeutic embolization, such as blindness or stroke.

Several publications focus on the potentially dangerous inferolateral trunk (ILT) of the cavernous internal carotid artery (ICA). The anatomy and neuro-angiography of the ILT have been described in detail. Some publications include photographs of detailed dissections of the human adult ILT [1–4]. No previously published photographs of a dissected fetal human ILT were found in the literature.

The presence of an embryologic persistent primitive maxillary artery (PPMA) is described in the literature dealing with the development of the ILT. There are differing concepts regarding the developmental origin of the ILT. It has been described as the PPMA [5]. Another concept postulated that this human embryologic branch of the cavernous ICA is the ILT [6, 7]. A third concept is that the ILT is a remnant of the lateral branch of the regressed primitive maxillary artery (PMA) [8]. In the articles discussing the above theories, the human embryologic vasculature is depicted only by schematic diagrams, without accompanying photographic images of the dissected embryologic inferolateral branch of the cavernous carotid. Thus, the review of the human fetal dissections in this investigation is relevant to the developmental and nomenclature issues of the ILT, PMA, and PPMA.

Certain developmental theories of the ILT postulate that this branch is homologous to the proximal part of the anastomotic artery (AA) in the dog. A number of cerebrovascular and neuroimaging publications describe hemodynamic investigations dealing with the unique features of the dog’s cranial circulation, focusing on the role of the maxillary artery (MA) anastomoses in the supply of the cerebral arterial circle.

Plain radiographic studies of the dog’s cranial circulation with limited cranial vascular visualization have been published [9–11]. Review of the literature found no high-resolution CT or CTA studies of the dog’s normal cranial circulation. An experimental study was published using a dynamic cone beam CT scan of the entire wash-in and wash out phases of a femoral vein injection in a dog [12]. The resulting images showed the major external carotid arterial branches. However, the cerebral arterial circle is barely visible and no anastomotic branches or other small branches are visualized.

A 3.0 Tesla and 7.0 Tesla TOF MRA study demonstrated a partially visualized cerebral arterial circle. The MA and its anastomotic communications with the partial visualized cerebral arterial circle were not demonstrated [13]. A study of ophthalmic artery variations in the human, included an image of the AA and its connections in a DSA of a dog [8].

The micro-CT images in this study (Figs. 3, 4, 5, and 6) show arterial branches smaller than 1 mm. Thus, micro-CT imaging of un-dissected arterially injected specimens, in several planes, using 3D volume images and multiple planar projections can provide precise identification of very-small-sized arterial anatomy.

## Materials and methods

### Examination of fetal material

During the years 1970–1973, fetal specimens were obtained for studies of the evolutionary and embryologic changes of the central nervous system, skull and spine. The specimens’ estimated fetal ages were determined by the crown-rump (CR) length and the occipito-frontal (OF) diameter [14, 15]. Some specimens were injected intra-arterially with a radiopaque barium-gelatin contrast agent. Arterial injections were performed manually under fluoroscopic control, by inserting a polyethylene catheter into the umbilical artery of the specimens. Following injection, fixation of the gelatin component of the contrast media was achieved by immersing the specimens in 10% buffered formalin for at least 10 days prior to dissection. Dissections were performed using a magnifying lamp and a dissecting microscope. Photography of the dissected specimens was performed.

### Examination of dog material

In the early 1970s, dog specimens, heparinized prior to euthanasia, were obtained, from a cardiac research laboratory. These specimens were injected intra-arterially with a radiopaque barium-gelatin contrast agent. Following injection, fixation of the gelatin component of the contrast media was achieved by immersing the specimens in 10% buffered formalin.

The dog specimens were kept in 10% buffered formalin until 1996 when the specimens were transferred to a solution of WARDSafe^™^ (Ward’s Science, Rochester, NY, USA), a specimen preservative solution. The specimens were kept in the WARDSafe^™^ solution until they were micro-CT scanned in 2010.

### Micro-CT imaging of the dog specimens

Due to the inability of CT techniques, available from the 1970s to the mid-2000s, to provide high-resolution imaging of the arterial circulation within the intact skull and brain, without dissection and histologic sectioning, no further imaging of the injected dogs was performed for many years. Only in 2010, a high-resolution micro-CT scanner became available with a bore large enough to scan the entire non-dissected head of the arterially injected dogs. Two of these dog specimens were scanned at Hadland Technologies, Amherst, NH, in a Nikon Metrology XTH225ST CT system, a high image resolution horizontal cone beam scanner with an amorphous silicon 16” × 16’’ Perkin Elmer 16 bit 2000 × 2000 pixel X-ray detector. Images were acquired using 135 kVp and 95 μA with 720 projections in 6 minutes. The images were reconstructed in 2.5 minutes with isotropic 89 μm voxels and saved in DICOM format.

## Results

### Dissection of Inferolateral branches of the cavernous carotid in human fetuses

**Fig. 1:**
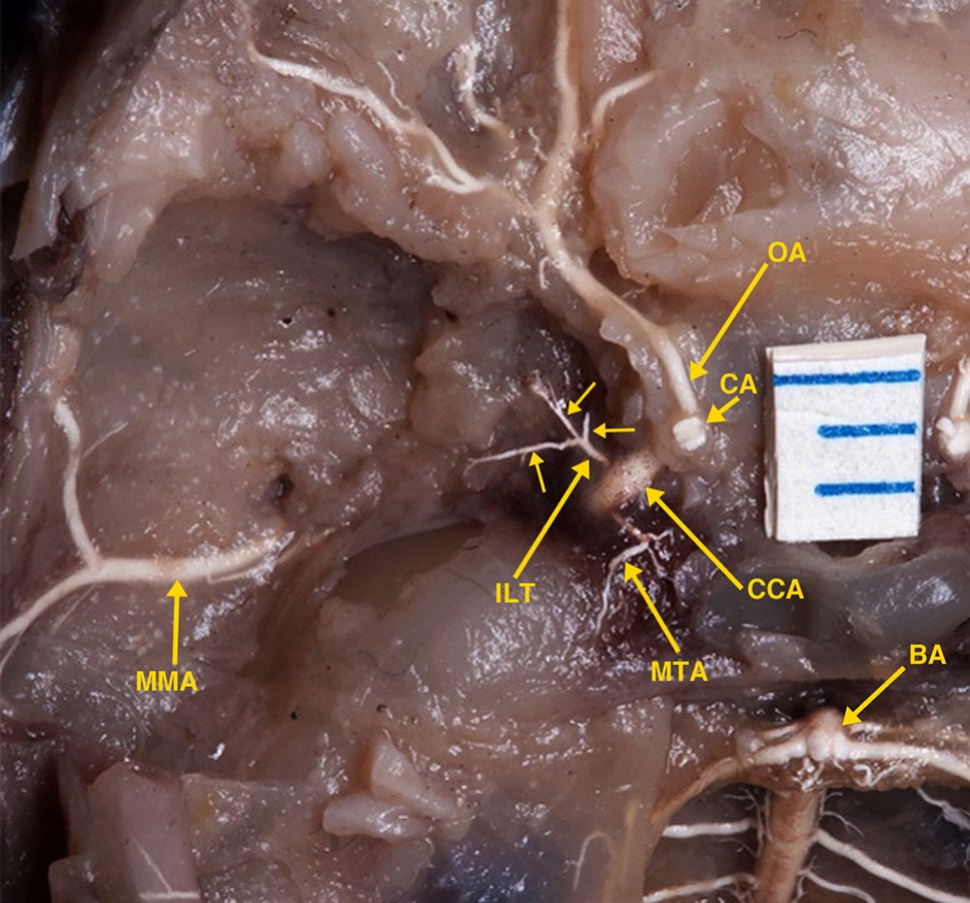
Photograph of a superior view of a dissected fetal skull base of a 14 weeks’ gestational age specimen, with a crown-rump length of 100 mm and occipito-frontal diameter of 40 mm, and arterially injected with a barium-gelatin contrast agent. The dissection demonstrates the inferolateral trunk (ILT), originating from lateral wall of the cavernous carotid artery (CCA) with its three branches. Also, dissected are the ophthalmic artery (OA) originating from the carotid artery (CA) at the anterior end of the cavernous carotid; the medial tentorial artery (MTA) originating from medial wall of the cavernous carotid artery; the middle meningeal artery (MMA); and the basilar artery (BA) and its branches. The distance between two blue lines on the ruler is 1 mm

Dissections of the cavernous carotid and the ophthalmic artery in a 14-week human fetal specimen (Fig. 1) demonstrated an arterial branch originating from the lateral wall of the cavernous carotid artery. This arterial branch artery is divided into small three branches with some similarity to photographs of dissections of the adult ILT in the literature [1–4]. A second lateral branch originating from the lateral wall of the cavernous carotid artery was not present in the dissected specimen.

**Fig. 2:**
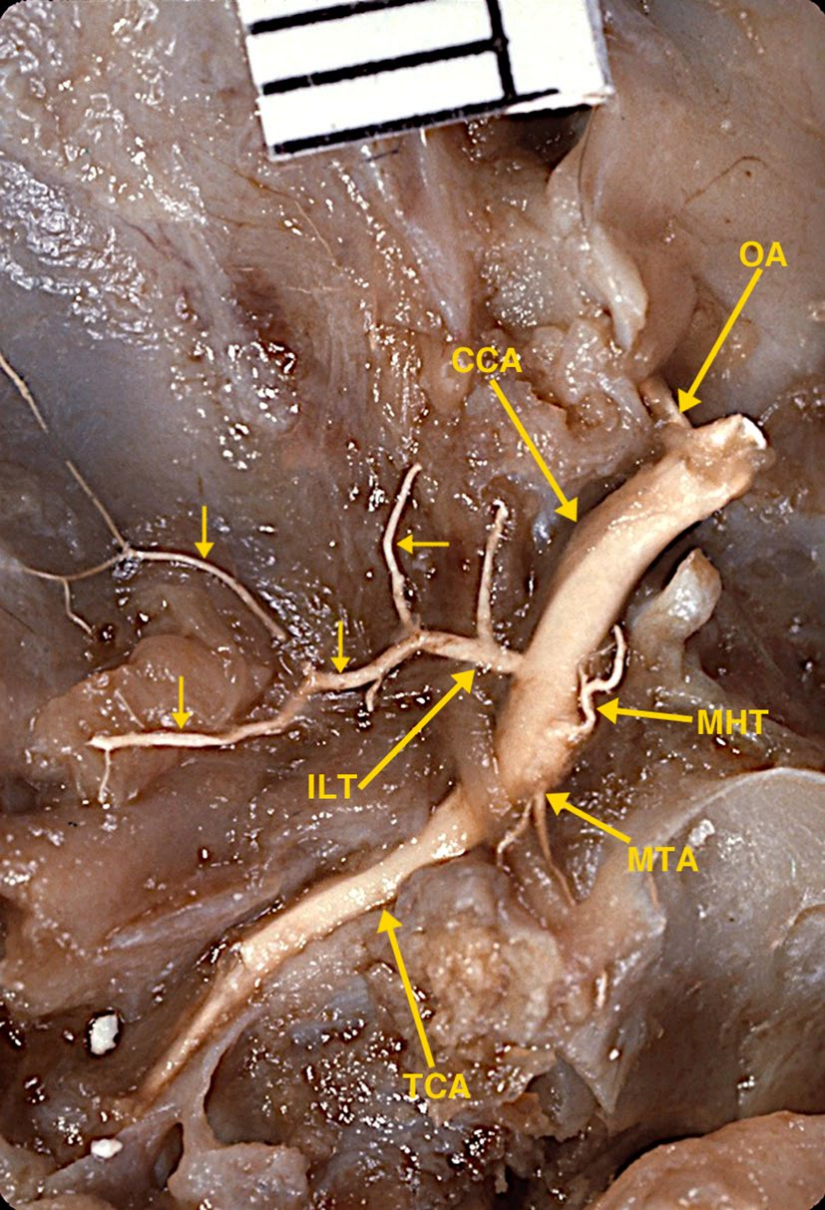
Photograph of a superior view of a dissected fetal skull base of a 24-week gestational age specimen, with a crown-rump length of 230 mm and occipito-frontal diameter of 85 mm, and arterially injected with a barium-gelatin contrast agent. The dissection demonstrates a larger inferolateral trunk (ILT) originating from lateral wall of the cavernous carotid artery (CCA) with its multiple branches. Also dissected are the origin of the ophthalmic artery (OA); the medial tentorial artery (MTA) and the meningo-hypophyseal trunk (MHT) originating from the medial wall of the cavernous carotid artery; and the temporal segment of the internal carotid artery (TCA). The distance between 2 black lines on the ruler is 1 mm

Dissection of the cavernous region of a 24-week human fetal specimen (Fig. 2) demonstrated a larger branch originating from the lateral wall of the cavernous carotid artery, with multiple branches extending along the floor of the middle cranial fossa, again with some similarity to photographs of dissections of the adult ILT in the literature [1–4]. A second lateral branch originating from the lateral wall of the cavernous carotid artery was not present in the dissected specimen.

### Processing the dog’s high-resolution micro-CT data

The DICOM data set was analyzed on an Apple laptop computer, using the OsiriX MD image processing software.[16] OsiriX MD is image processing and visualization software dedicated to DICOM images. The software runs on Apple OS X systems, providing post-processing functions, such as 3D volume rendering and multi-planar reconstruction, with the ability to change the plane angulation, slab thickness, and delete bony and soft tissue structures from the data set. These technical features permitted more precise identification of specific arterial anatomy.

### Vascular anatomy of the dog obtained by the Osirix MD software

**Fig. 3:**
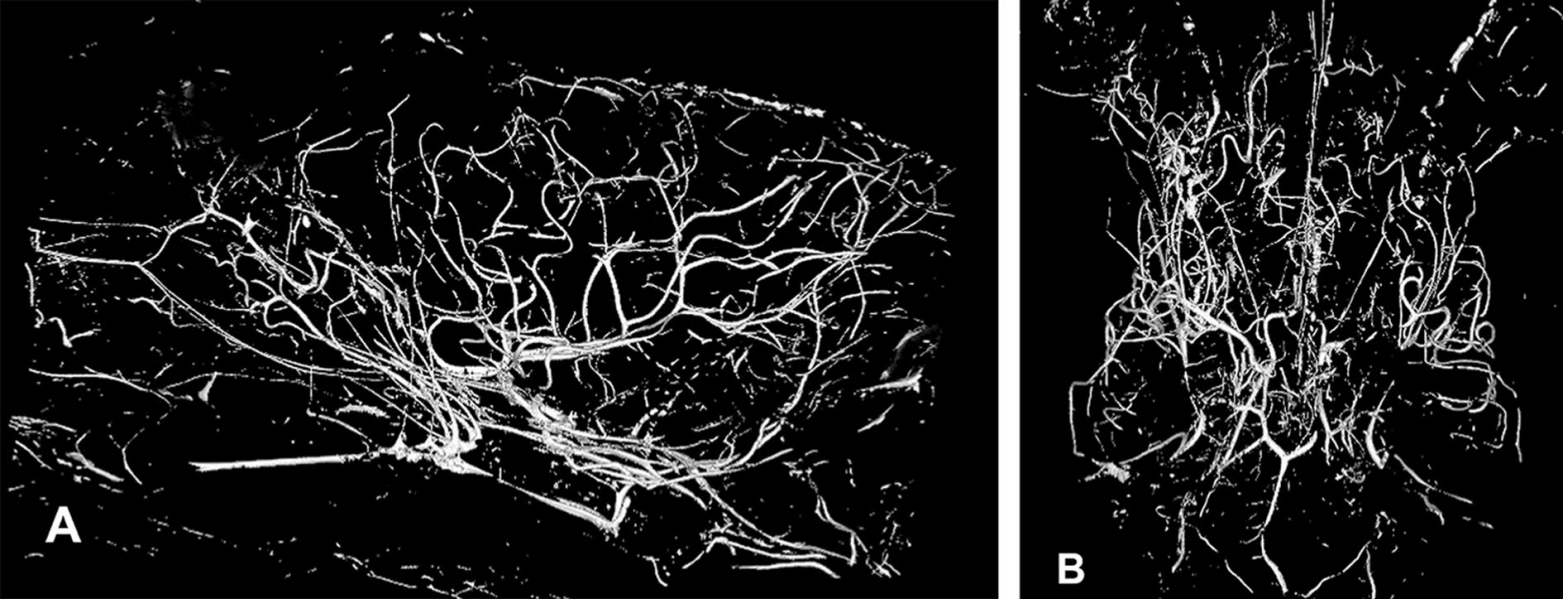
Lateral (**A**) and dorsoventral (**B**) 3D volume rendered micro-CT projections of the cranial arterial circulation of a dog. Of note is the extent of the demonstrated vascularity in the bone and soft tissue free images. These 3D images can be rotated and turned in any direction or angle needed. In view of the extensive vascularity, it is difficult to identify the anastomotic artery and the ramus anastomoticus and their relationships to other arteries

**Fig. 4:**
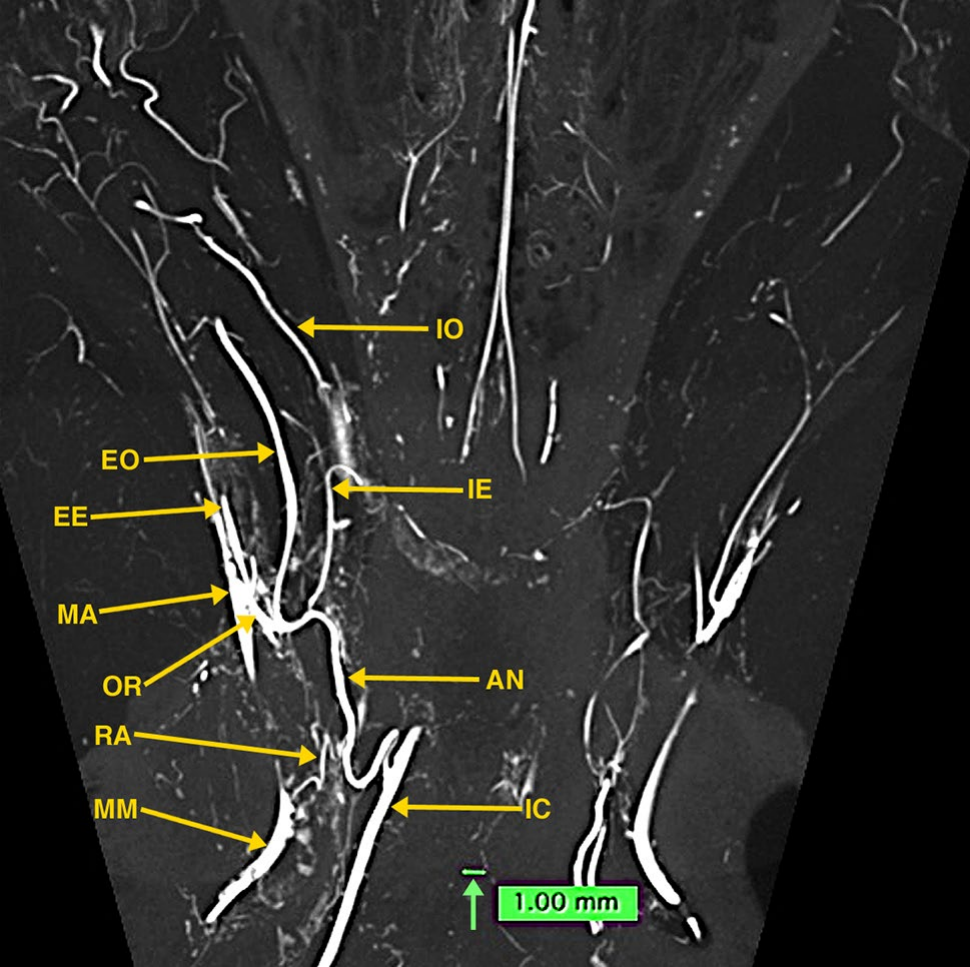
A 10-mm thick oblique dorsoventral multi-planar reconstruction (MPR) micro-CT image of a dog, using maximum intensity projection (MIP). The short orbital artery (OR) arising from the maxillary artery (MA) gives rise to the anastomotic (AN), external ophthalmic (EO), and external ethmoidal (EE) arteries. The anastomotic artery, joined by the ramus anastomoticus (RA), a branch of the middle meningeal artery (MM) connects with the internal carotid artery (IC), thus forming the major anastomotic connection between the internal and external carotid circulations. Also, identified in this thin slab are segments of the internal ophthalmic (IO) and internal ethmoidal (IE) arteries which are branches of the anterior cerebral artery (AC) as seen in Fig. 5. A green-colored 1.0 mm scale bar (green arrow) in Figs. 4, 5, 6 highlights the small size of some of the visualized blood vessels

**Fig. 5.**
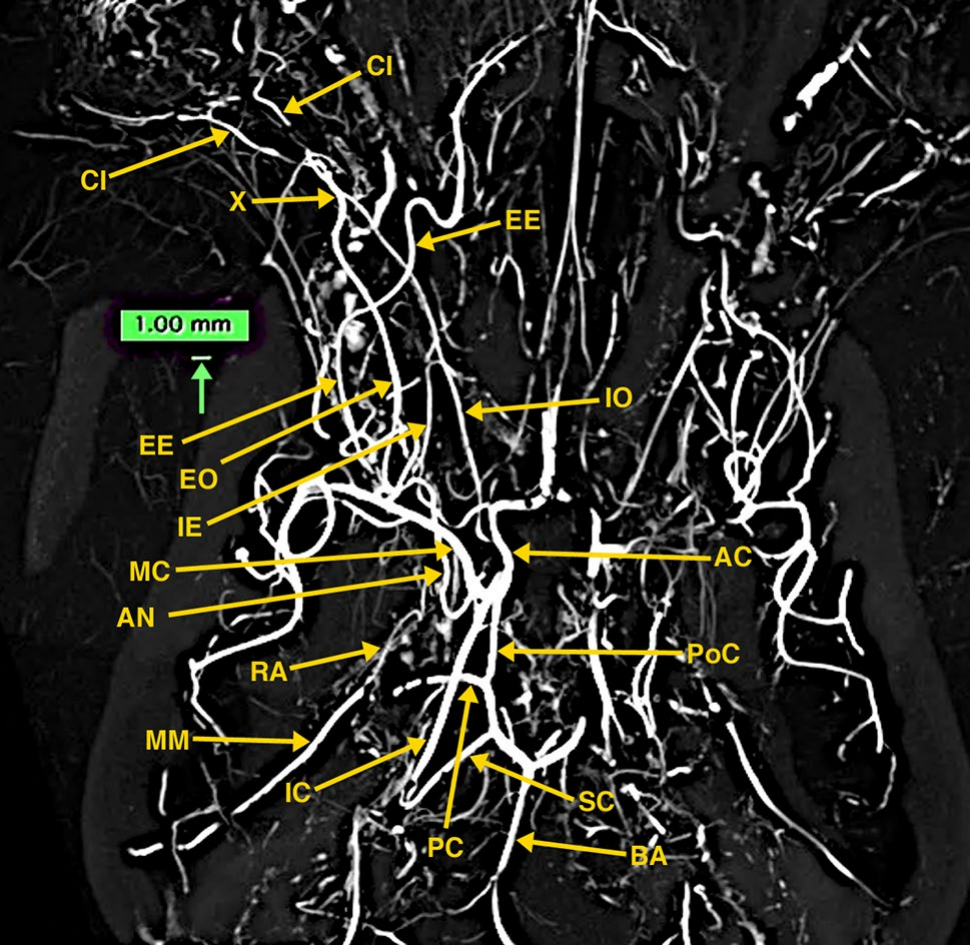
20 mm thick oblique dorso-ventral multi-planar reconstruction (MPR) of a micro-CT image of a dog, using maximum intensity projection (MIP). In this thicker slab, some structures partly visible in Fig. 4 are easier to identify. The origin of the internal ophthalmic (IO) and the internal ethmoidal (IE) medial to it, both arising from the anterior cerebral artery (AC) are identified. The anterior cerebral and the middle cerebral (MC), posterior communicating (PoC), posterior cerebral (PC), superior (anterior) cerebellar (SC), basilar (BA) and internal carotid (IC) arteries form the cerebral arterial circle. The anastomotic site (X) of the external (EO) and internal (IO) ophthalmic arteries and the ciliary arteries (CI) are identified. A light blue-colored 1.0 mm scale bar (green arrow) highlights the small size of some of the visualized blood vessels

**Fig. 6.**
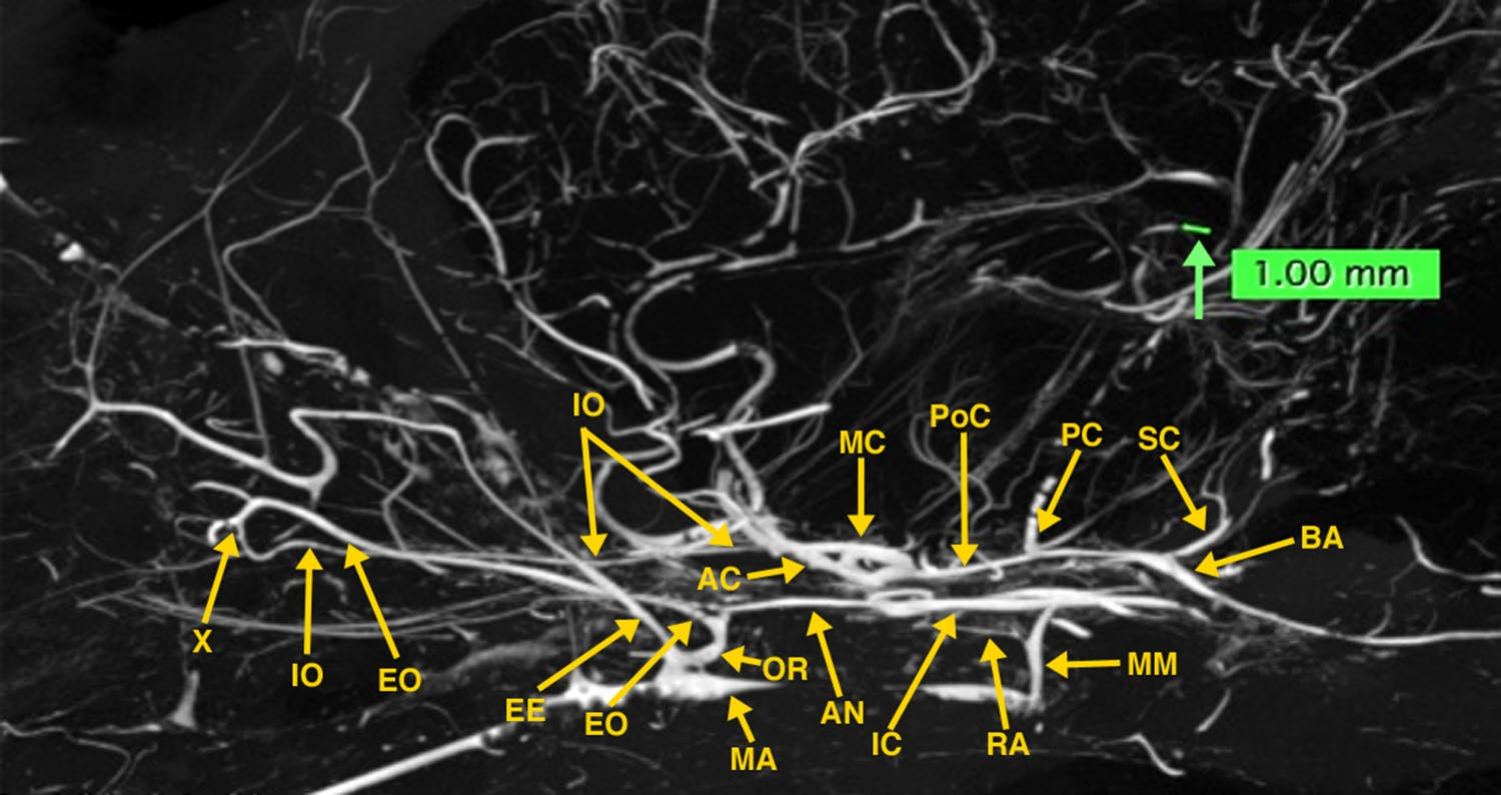
20 mm thick sagittal multi-planar reconstruction (MPR) of a micro-CT image of a dog, using maximum intensity projection (MIP). In this view, the short orbital artery (OR) arising from the maxillary artery (MA) gives rise to anastomotic (AN), external ophthalmic (EO), and external ethmoidal (EE) arteries. The anastomotic artery (AN), joined by the ramus anastomoticus (RA), a branch of the middle meningeal artery (MM) connects with the internal carotid artery (IC). The internal ophthalmic artery (IO) arising from the anterior cerebral artery (AC) is identified passing anteriorly to anastomose (X) with the external ophthalmic artery. The middle cerebral (MC), posterior communicating (PoC), posterior cerebral (PC), superior (anterior) cerebellar (SC) and basilar (BA) are identified

The obtained high-resolution micro-CT images (Figs. 3, 4, 5, and 6) demonstrated, without the presence of bone and soft tissue, high-resolution vascular anatomy of the maxillocarotid and middle meningeal anastomotic arteries. Also, visualized were the orbital, external and internal ophthalmic, external and internal ethmoidal, and other skull base and brain arteries (Figs. 4, 5, and 6). The 1.00 mm scale bars in Figs. 4, 5, and 6 point out the sub-mm size of many of the visualized arterial structures.

## Discussion

In view of the different theories regarding the developmental links of the ILT of the human cavernous ICA and the dog’s cranial vasculature, the discussion will start with a review of certain aspects of the dog’s orbitocranial arterial circulation.

### The anastomotic orbitocranial arterial circulation of the dog

The maxillocarotid anastomotic artery and middle meningeal anastomotic artery of the dog are communications between the maxillary and the internal carotid arteries that are considered to be a major blood supply to the cerebral arterial circle. In the literature, the maxillocarotid anastomotic artery is frequently just named the anastomotic artery (AA), and the middle meningeal anastomotic artery is named the ramus anastomoticus (RA). In view of the multiple cranial anastomoses between the ICA and the external carotid artery, De La Torre et al. [10] preferred using the terms maxillocarotid anastomotic artery instead of the AA.

The MA, after crossing the mandibular division of the trigeminal nerve, gives off the RA which enters the cranium through the foramen ovale and, after giving rise to the middle meningeal artery (MM) anastomoses with the AA in the cavernous sinus [9, 11].

Detailed canine dissection diagrams [17], and textbook color illustrations [18] describe that the MA, a short distance after passing into the floor of the orbit, becomes the infraorbital artery, which after a short distance gives rise to the orbital artery. The orbital artery immediately divides into the AA, external ophthalmic, and external ethmoidal arteries as demonstrated in the multi-planar reconstructions of the micro-CT images of the dog specimen (Figs. 4 and 6). These three arteries may also arise directly from the MA. The AA may also arise from the external ophthalmic artery. It passes dorso-medially within the cavernous sinus and the sinus extension within the superior orbital fissure. Within the cavernous sinus, it is joined with the RA of the MM before connecting with the ICA (Fig. 4). The origin of external ophthalmic artery is also variable. It runs along the dorsal surface of the optic nerve where it anastomoses with the smaller internal ophthalmic artery (Fig. 5). The internal ophthalmic artery originates from the anterior cerebral artery lateral to the origin of the internal ethmoidal artery (Fig. 5). The cerebral arterial circle is formed by the internal carotid, and the anterior cerebral, middle cerebral, posterior communicating, posterior cerebral, and superior cerebellar arteries.

The AA and the RA have been described not only in the dog but also in the cat, sheep, goat, ox, and minimally in the pig [9], as well in the raccoon, wolf, badger, and civet [19]. It appears that in this group of mammals, a significant part of their cerebral blood flow to the cerebral arterial circle is transmitted from the maxillary artery via the AA and the RA [20]. Studies show that the anastomotic blood flow is about half of the ICA flow [19, 21].

### Theories regarding evolutionary links between human and dog orbitocranial arterial anastomoses

There are several theories in the literature in regards to the possible evolutionary links between the anastomotic cranial blood vessels of the dog, and the human carotid branches, such as the PMA, the PPMA, and the ILT.

### Primitive maxillary artery (PMA)

According to Moffat [22], Sabin [23] was the first to use the term PMA in one of her figures of a pig embryo. Sabin’s article shows in plate VII a drawing of the vascularity of a pig embryo with a small cluster of vessels and the annotation a.m.p for ‘arteria maxillaris primitiva’.

Moffat [22] in a study of rat embryos noted that at the 2–4 mm embryo stage, the primitive ICA is already present and gives off a prominent cluster of PMA arterioles, supplying the forebrain and the optic stalk. Already at the 6–9 mm embryo stage, the PMA is starting to diminish in size and only its proximal part is occasionally identified.

Padget [24] in her study of the arterial development of the human embryo stated that the stem of the transitory ophthalmic artery persists in a reduced form and supplies the medial parts of the maxillary region around Rathke's pouch. Padget named this artery the primitive maxillary artery. As Moffat [22] pointed out, Padget shows a reconstructed image of the PMA in Padget's Fig. 3. The name PMA appears in Padget's Fig. 12, a composite diagram, as one of the four names given by different authors to a tiny stem. She described that this artery undergoes a functional change. At the 5 mm stage, it supplies the optic region. At the 18 mm stage, the appearance of the permanent stem of the ophthalmic artery confines the stem of the primitive maxillary artery to the cavernous sinus, where it participates in the supply of the infundibular process of the hypophysis. Padget stated that the primitive maxillary artery becomes incorporated in the formation of the inferior hypophyseal artery, a component of the meningo-hypophyseal trunk.

Thus, Padget is clear in noting that at a very early developmental stage, the PMA becomes incorporated in the formation of inferior hypophyseal artery, and as Moffat [22] notes, by the 12 mm stage, the PMA could not be always identified. Padget’s description is also mentioned by Lasjaunias and Bernstein [1] and by De La Torre et al. [10]. The latter authors state that “the hypophyseal artery in both the dog and man, was the closest evolutionary link of the dog’s maxillo-carotid anastomotic artery.”

Gregg et al. [8] mention that after the 5–6 mm stage, the lateral branch of the PMA regresses and its remnant form, the recurrent ophthalmic artery (OA) and the ILT, referencing Padget [24] and De la Torre and Netsky [5]. These authors do not mention the recurrent OA and the ILT as arising from the PMA.

### Persistent primitive maxillary artery (PPMA)

The human PPMA is a puzzling arterial structure, resulting from the lack of photographic evidence of a dissection of a human PMA and PPMA in the literature.

De La Torre and Netsky [5] in their frequently cited publication, studied the similarities of the cranial circulation of human and the dog [5]. When performing dissections on human fetal specimens with estimated fetal age ranging from 5 to 9 months, they noted that from the cavernous ICA, at the level of the medially directed posterior inferior hypophyseal artery originated a laterally directed branch, “previously not described”. *They identified no other laterally directed branches arising from the cavernous ICA.* They named this lateral branch the persistent PMA (PPMA). This artery had anterior, lateral and posterior branches. Unfortunately, they did not include a photograph of their dissection of the PPMA. According to their article, the PPMA in adult man remains as a lateral meningeal branch of the ICA. They also stated that the lateral branch of the PPMA in the human fetus is the homologue of the proximal segment of the maxillocarotid anastomotic artery in the dog, and that the maxillocarotid anastomotic artery embryologically develops from the PMA. They also stated that the PPMA in the adult human is homologous to the segment of the anastomotic artery near the carotid in dog.

Both fetal specimens dissected in this investigation showed inferolateral branches of the cavernous ICA. Dissection of the 14-week-old specimen (Fig. 1) demonstrated the inferolateral trunk (ILT), originating from the lateral wall of the cavernous carotid artery with three branches. Dissection of the 24-week-old specimen demonstrates a larger inferolateral trunk (ILT) originating from the lateral wall of the cavernous carotid artery with multiple branches.

Again, it is important to note that De La Torre and Netsky [5] stated that they *saw no other lateral cavernous carotid branch except for the PPMA*. In view that the dissected specimen in Fig. 1 in this investigation was of a younger developmental stage, and the lack of photographs of their dissected specimens, one has to conclude that what De La Torre and Netsky described as the PPMA was actually the ILT.

It is important to note that all the subsequent publications discussing the PPMA rely only on the study of De La Torre and Netsky [5] without citing any other confirmatory evidence.

Toma, in reviewing the embryology of the OA [25], seemed to express concerns about the PPMA. In a schematic diagram of potential anastomoses between the OA and MA, he depicts the ILT and the PPMA as a single vessel. He notes that “as the primitive OA develops, the PMA dwindles in size, and the only remnant becomes the inferior hypophyseal artery. The discrepancy between the early and late stages of the PMA and PPMA remain uncertain and further research is warranted to corroborate the fate of the primitive arteries.” His concerns about an artery, such as the PMA dwindling and then reappearing as the PPMA, are resolved by this investigation that determined that the ILT was misidentified as the PPMA, the latter being a non-existent anatomic structure.

Also of note are several publications [1, 4, 26–28] that describe the PMA as originating from the C5 segment of the future carotid siphon and supplying the posterior hypophysis. The PMA may give rise to a meningeal branch to the dorsum sellae or as a common trunk with the trigeminal artery. These authors [1, 4, 26–28] describe the PMA as distinct from the ILT that originates from the C4 segment of the future carotid siphon and follow Padget’s description of the PMA terminating in the posterior hypophyseal artery.

### Homologies of the anastomotic artery (AA) of the dog and the inferolateral trunk of the human cavernous carotid artery (ILT)

Lasjaunias et al. [6, 7], when comparing the dog’s and the human embryo’s cavernous region with diagrams, postulate that the ILT of the cavernous carotid artery in the human may be in part homologous to the proximal part of the anastomotic artery in the dog and that the embryonic vidian and stapedial arteries of man are the distal anastomotic artery homologues. These authors [6, 7] note that homologies are better appreciated in the human fetus than in the adult in whom many branches have dwindled considerably.

Lasjaunias et al. [7] in a diagrammatic illustration outlining the recurrent cavernous branches of the OA note that in the dog an anastomotic artery passes through the superior orbital fissure from the carotid siphon and anastomoses with the internal OA, a branch of the ACA. As pointed out in illustrations by Miller et al. [18] and De La Torre and Netsky [5], the anastomotic artery connects the ICA with both the IO and EO as also shown in the dog’s angiographic image by Gregg’s et al. [8] and in Figs. 4, 5, and 6 of the high-resolution micro-CTs in this investigation.

Lasjaunias et al. [6, 29] when discussing the ILT, note that ILT corresponds to the proximal (carotid) segment of the embryonic dorsal OA and the distal segment of the dorsal OA corresponds to the deep recurrent OA. At the 12 mm stage, the human embryo has 2 OAs. The ventral one arises from the ACA and the dorsal one from the carotid siphon. The authors note that this is homologous to the dog where the homologue of the dorsal artery of the human is the anastomotic artery.

Gregg et al. [8] note that Padget [24] never mentioned that the primitive dorsal OA artery may arise from the cavernous carotid, via the ILT, and that the description by Lasjaunias et al. [6] erroneously describe that the ILT and the deep recurrent OA are adult remnants of a primitive dorsal OA. Gregg et al. [8] state the ILT and the deep recurrent OA are developmentally linked to the lateral branch of the PMA.

Daniel et al. [9] in their extensive study of the carotid rete suggested that the anastomotic artery and the ramus anastomoticus are homologous, respectively, with the recurrent meningeal and middle meningeal arteries of the rabbit and man. Daniel et al. also state that the anastomotic artery could be considered as a modified supra orbital division of the stapedial artery in view of its passage through the superior orbital fissure accompanying the ophthalmic division of the trigeminal nerve and its relationship with the external OA. This phylogenetic association suggests that these arterial variations are due to modification of the stapedial artery at an earlier stage.

Lasjaunias et al. [30] include a fetal specimen of unstated age showing an injected MM and meningeal twig extending from the region of the cavernous sinus to the LA. In the text of their article it is stated “The deep recurrent OA arises from the first part of the intra-orbital portion of the OA and then joins the cavernous sinus through the sphenoidal fissure in the Zinn’s tendon (it is the residual artery of the dorsal artery of the human embryo and of the anastomotic artery of the dog”.

## Conclusion

This investigation provided previously unavailable photographs of dissected human fetal inferolateral trunk of the cavernous carotid artery, but not a persistent primitive maxillary artery. The persistent primitive maxillary artery is a non-existent anatomic structure that likely was a previously misidentified cavernous carotid inferolateral trunk.

Micro-CT images of the dog provided high-resolution vascular anatomy of the maxillocarotid and the middle meningeal anastomotic arteries, and their connections with the internal carotid, maxillary, and middle meningeal arteries. Also, visualized were the orbital, external and internal ophthalmic, external and internal ethmoidal, and other skull base and brain arteries. All these features were not previously visualized with computed tomography.

This investigation contributes to the understanding of the phylogenetic and the embryologic anatomy of the region of the branches of the intra-cavernous internal carotid artery, a region for potential dangerous complications of intra-arterial therapeutic embolization by super-selective interventional neuro-angiography.

## Abbreviations

AA: Anastomotic artery
ICA: Internal carotid artery
ILT: Inferolateral trunk of the cavernous internal carotid artery
MA: Maxillary artery
MM: Middle meningeal artery
OA: Ophthalmic artery
PMA: Primitive maxillary artery
PPMA: Persistent primitive maxillary artery
RA: Ramus anastomoticus
